# Interpreting the Association Between Diuretic Intensity Score and Mortality: The Potential Roles of Diuretic Responsiveness and Mineralocorticoid Receptor Antagonist Therapy

**DOI:** 10.1002/clc.70404

**Published:** 2026-06-30

**Authors:** Teruhiko Imamura

**Affiliations:** ^1^ Second Department of Internal Medicine University of Toyama Toyama Japan

**Keywords:** cardiorenal syndrome, congestion, heart failure

To Editor,

We read with great interest the article, which developed a novel Diuretic Intensity Score (DIS) and reported an inverse association between higher DIS and short‐term mortality among hospitalized patients with heart failure [1]. Several concerns have been raised.

The DIS reflects prescribed treatment intensity but does not account for actual diuretic responsiveness [1]. Patients receiving high‐intensity diuretic therapy may represent a subgroup with preserved natriuretic capacity and adequate renal reserve, allowing clinicians to continue escalating therapy. In contrast, patients with advanced cardio‐renal syndrome, hypotension, severe renal dysfunction, or marked diuretic resistance may be unable to tolerate intensive decongestive treatment despite having greater congestion severity. Previous studies have demonstrated that diuretic responsiveness, assessed by urine output, weight loss, or natriuresis per unit of loop diuretic administered, is strongly associated with clinical outcomes in acute heart failure [2]. Therefore, the favorable prognosis observed in patients with higher DIS may partly reflect preserved responsiveness to diuretics rather than a direct benefit of intensive diuretic exposure itself.

The construction of the DIS includes spironolactone as one of the score components [1]. Unlike loop and thiazide diuretics, spironolactone is not merely a decongestive agent but also a guideline‐directed disease‐modifying therapy with established prognostic benefits in selected heart failure populations, particularly those with reduced ejection fraction [3]. Consequently, a higher DIS may partly capture greater exposure to mineralocorticoid receptor antagonist therapy rather than greater diuretic intensity alone. This issue is particularly relevant because the score assigns additive value to combination therapy, potentially increasing DIS among patients receiving spironolactone‐containing regimens. Additional analyses excluding spironolactone from the scoring system or adjusting for mineralocorticoid receptor antagonist use separately may help clarify whether the observed association is attributable to intensified decongestion, disease‐modifying pharmacotherapy, or both.

## Funding

The author has nothing to report.

## Ethics Statement

The author has nothing to report.

## Consent

The author has nothing to report.

## Conflicts of Interest

The author declares no conflicts of interest.

## Data Availability

The author has nothing to report.
